# Provision of dementia-related services in Canada: a comparative study

**DOI:** 10.1186/s12913-016-1435-1

**Published:** 2016-05-17

**Authors:** Helen Tam-Tham, Alberto Nettel-Aguirre, James Silvius, William Dalziel, Linda Garcia, Frank Molnar, Neil Drummond

**Affiliations:** Department of Community Health Sciences, University of Calgary, Calgary, Alberta Canada; Department of Paediatrics, University of Calgary, Calgary, Alberta Canada; Department of Medicine, University of Calgary, Calgary, Alberta Canada; Department of Medicine, University of Ottawa, Ottawa, Ontario Canada; Interdisciplinary School of Health Sciences, University of Ottawa, Ottawa, Ontario Canada; Bruyère Research Institute, Bruyère Continuing Care, Ottawa, Ontario Canada; Department of Family Medicine, University of Alberta, Edmonton, Alberta Canada

**Keywords:** Health services delivery, Dementia, Vignette, Canada

## Abstract

**Background:**

Dementia is common, particularly among older adults, and is one of the major causes of dependency later in life. We sought to provide an overview and comparison of key services related to dementia care as the disease progresses in three large Canadian healthcare centres.

**Methods:**

We identified family physicians, geriatric specialists, and dementia case managers from three major population centres in Canada with universal healthcare coverage. Using a standardized longitudinal dementia case vignette, participants were interviewed on services they would provide at each stage of the disease. We used principles of content analysis to generate codes and identify themes; appropriate time frames from the vignette fitting the necessary provision of services were derived from the Canadian consensus statement and determined in consultation with clinical experts. Proportions of participants that identified dementia-related care services were analyzed at each time point of the vignette using chi-square tests.

**Results:**

Thirty-four healthcare providers from Calgary (Alberta), Edmonton (Alberta), and Ottawa (Ontario) participated. Review of our data identified seven overarching themes of dementia-related care services. Services provided in the community setting include future planning and related services, educational and social support services, and home care and respite services. Although all providers consistently identified educational and social support services (e.g. the Alzheimer Society) within the appropriate time frame, the provision of other services was variable. The proportion of providers reporting potential access of future planning services was significantly different across the three sites (Calgary, 91.7 %; Edmonton; 58.3 %; and Ottawa, 30.0 %), *p* = 0.012. Also, the proportion of providers that identified day program services were significantly different across the three sites (Calgary, 100.0 %; Edmonton, 91.7 %; and Ottawa, 60.0 %), *p* = 0.023 according to a chi-square test.

**Conclusions:**

We found important types of variability in service delivery among different regions in Canada for a typical patient with dementia and their family caregiver. Health systems can be calibrated by aligning services from different settings to appropriate time points in the vignette, which illustrates the dynamic course of service delivery and opportunities for improvement throughout the disease trajectory.

**Electronic supplementary material:**

The online version of this article (doi:10.1186/s12913-016-1435-1) contains supplementary material, which is available to authorized users.

## Background

Approximately 24 million people aged 60 years or older live with dementia across the globe, 40 % of which reside in higher income settings such as Canada [1, 2]. Dementia is generally progressive, life-limiting, and one of the major causes of dependency later in life [3]. Approximately 7.7 million new cases of dementia are reported each year; a new case every four seconds [2]. Although there are many causes of dementia, the disease is strongly (though not exclusively) associated with an elderly population. Alzheimer’s disease, vascular dementia, and mixed dementia (exhibiting elements of Alzheimer’s and vascular dementia) represent the great majority of dementias in community settings [4]. In Canada, about 62 % of all dementias are identified as Alzheimer’s disease, 12 % are vascular dementia, and a further 13 % are identified as mixed Alzheimer’s and vascular dementia [4].

With the appropriate support, people with dementia can continue to contribute in society and maintain a positive quality of life. However, many people with dementia tend to depend on their family caregivers to remain in the community. In Canada, up to 90 % of in-home care is provided by family caregivers for people with dementia [5]. Although various sources of support including community services aim to fulfill the daily necessities of this client population and Canadians with dementia indicate that support services are indispensable [6], there are challenges to their availability, delivery, and uptake [7, 8], which places more pressure on family members, other unpaid caregivers, use of private agencies, and institutionalization.

In Canada, people with dementia have access to healthcare services from their health insurance implemented through their provincial government. However, availability of services is unique to each community and it is difficult to ensure quality of care due to the complexity of patient needs, limited capacity of support services, and inadequate inter-organizational coordination [9]. The types of local and national services provided for each stage of dementia have not been thoroughly investigated in settings across Canada, and doing so may provide insight for a national approach to support people living or caring for those with this mental disorder.

### Aim

We evaluated the provision of services among healthcare providers in three large Canadian population centres in terms of its concordance with recommendations from the Canadian consensus statement on the diagnosis and treatment of dementia [10] regarding *what* services would be recommended to be accessed and *when* based on a generic dementia disease trajectory as described in a case vignette.

## Methods

### Participant selection and practice setting

Healthcare providers in Calgary (Alberta), Edmonton (Alberta), and Ottawa (Ontario) were purposively selected to capture a range of clinical roles and circumstances. Invitations were mailed or faxed to providers identified from public directories available from major organizations in each region (e.g. medical centres, seniors health clinics, and home care programs). Providers known to the investigators were also invited and participants could recommend others who could offer a different perspective about dementia-related services. Only providers caring for patients with dementia with at least one year of experience in their respective fields were eligible to participate. Interviews were conducted in clinic offices and via telephone (see Additional file 1 for interview guide). The participants were provided with a vignette [11] at least one week in advance of their interview.

### Development of a case vignette

The vignette (Table 1) was developed to be representative of a typical presentation of dementia in community-based primary care settings (i.e. expressing symptoms of Alzheimer’s disease, vascular dementia, and mixed Alzheimer’s and vascular dementia). It was developed to contribute to building a regionalized dementia network in eastern Ontario, Canada, with a focus on phrases of transition for improved patient-centered care [11]. It was also developed to provide a framework for medical education regarding transition points for patients with dementia. The vignette was developed by an expert advisory committee consisting of a family caregiver, clinicians, and representatives from key organizations providing services for people with dementia and their family caregivers. The vignette illustrated 13 time points from mild cognitive impairment (i.e. ≤ time point 4) to a moderate dementia stage (i.e. time points 5 to 8) and later/severe dementia stages (i.e. ≥ time point 8) of the disease trajectory.

**Table 1:** The dementia case vignette

Time	Description
Medical background	Mrs. G.C. is a 76 year old married woman with Grade 12 education. She had a mother who developed Alzheimer’s Disease onset age 84. Her medical history including hypertension, hyperlipidemia and osteoporosis. Her medications are Hydrochlorothiazide, Adalat XL, Lipitor, Calcium, Vitamin D, and Fosamax.
0 months (T1)	*Warning signs*
	In the last six months her husband noted that she seemed to be a little bit forgetful, having some problems with names, “not quite as sharp” as one year previously, having a little more difficulty planning the bigger family social events and being a little less interested in leisure activities. She was still driving, shopping, cooking, independent in all her IADL’s although she occasionally needed a reminder to take her medication.
6 months (T2)	*Screening results and early recognition*
	While at the local Pharmacy her husband noticed that the Pharmacist was offering a 2 min Dementia Screening Test so he and Mrs. G.C. did the test. He was fine but his wife had difficulties in animal naming (9 in one minute) and clock drawing. He realized that this was a significant issue which needed medical attention.
7 months (T3)	*Mild cognitive impairment*
	Her husband was now worried that this was more than normal ageing and did in fact arrange an appointment with the family physician.
	The family physician tested first with the MMSE on which her score was 25/30. Laboratory testing was negative. The conceptualization was that Mrs. G.C. was not as “sharp with her memory” as she was six months previously but no other areas of cognitive function or functional abilities were affected.
	The Family Physician explained the concepts of mild cognitive impairment and gave advice about being physically, mentally and socially active. He explained that it could progress to more problems with memory and said that he would see her in one year or earlier if there was greater concern about memory or function. The patient’s hypertension and hyperlipidemia were well controlled and enteric coated aspirin was started at 81 mg daily.
1 year and 7 months (T4)	*Annual follow-up*
	One year later there didn’t seem to be any progression of symptoms or functional loss. Her MMSE was now 24/30.
2 years and 7 months (T5)	*Diagnosis*
	One year later the husband was more concerned because she got lost once while out driving the car back from her sister’s home 30 miles away, and because he noticed that she was having more trouble with cooking more complicated meals, being more forgetful about medications and occasionally having angry outbursts. He was a little bit worried about leaving her alone for a weekend to go to his big curling bonspiels in the winter. Her MMSE was now 20/30. Her family physician did further evaluation which showed poor visual spatial function (clock drawing) and poor performance of Trails A and Trails B.
	A CT scan was done which showed periventricular white matter changes and two old lacunar infarcts. The family physician made the diagnosis of mild mixed Alzheimer’s and vascular dementia and she was started on cholinesterase inhibitor treatment. Based on her overall assessment he advised her that she needed to stop driving.
2 years and 10 months (T6)	*3 month follow-up*
	Three months later she was seen and she had improved. She was more active, more attune to social situations and conversation and more like her old self. Her MMSE had improved to 22. At this stage she only needed a little bit of cueing for finances and shopping.
	She was referred to a Day Centre at a Senior’s Centre for increased stimulation and socialization and to provide her husband with some respite.
3 years and 7 months (T7)	*Increased support for IADL*
	Nine months later she was about the same, though a little more forgetful. Her husband had hired a maid to do some of the simple cleaning services through the local community for-profit support agency and he also needed to become more involved in cooking simple meals, shopping and finances. Her MMSE was now 20/30.
4 years and 7 months (T8)	*IADL, ADL, and behavioral and psychological symptoms*
	One year later she was more forgetful, was unable to cook on the stove but still could use the microwave and do simple cold meals. She needed help with laundry and help with shopping. She was independent in her personal ADL’s and only occasionally needed some cueing with respect to clothes selection. She did need help with respect to bathing and homecare became involved. Her MMSE was 16/30. She was more emotionally labile, apathetic and became very anxious if left alone. She was also having episodic bouts of agitation and occasionally aggressive behavior.
	Memantine (Ebixa) was started and there was some improvement in terms of cognition, (MMSE 18/30), ADL, agitation and anxiety.
5 years and 7 months (T9)	*Increased support in ADL and caregiver stress*
	One year later her MMSE had declined to 15/30. Her husband was doing all the instrumental activities of daily living. She needed help with bathing, hygiene and toileting and there was considerable caregiver stress in that she could only be left alone for approximately an hour. Homecare was providing more services in terms of bathing and personal care. She was occasionally incontinent. Her gait was unsteady and her fall risk was increased, and thus she needed to use a walker.
	A day program helped with respect to daytime respite and there was an increase in paid services by the husband to lessen caregiver stress.
6 years and 7 months (T10)	*Stroke and hospitalization*
	One year later she had a small stroke leaving her with some weakness on the right side. Her incontinence was worse. She was admitted to the hospital where she became much more confused.
6 years and 8 months (T11)	*Transition from home to long-term care*
	Following a conference attended by phone by their daughter in Florida who felt that her parents should be together, she was discharged home. She developed a tendency towards wandering about the house and once wandered outside. Her husband was no longer able to look after her. It was decided that she would re-locate to residential care. This move was very positive for the husband.
7 years and 8 months (T12)	*Increase in behavioral and psychological symptoms*
	One year later her communication skills were markedly affected. Her mobility was decreased. The nursing staff noted that she began having increasing hallucinations and angry outbursts.
8 years and 8 months (T13)	*Death*
	One year later after receiving appropriate end of life care she was found deceased on morning nursing rounds.

*ADL* activities of daily living, *IADL* instrumental activities of daily living, *MMSE* mini-mental state examination, *T* time point. Adopted from [11]

### Data collection

Research coordinators from each site conducted structured face-to-face or telephone interviews with each participant between January and April 2009. Participant recruitment ceased when theoretical saturation was reached. All interviews were audio-recorded and transcribed verbatim. Written or verbal consent for participation in the study was obtained from participants prior to the interview. This study was approved by the Conjoint Health Research Ethics Board of the University of Calgary and the Council of Research Ethics Board of the University of Ottawa.

### Analysis

The transcripts were coded manually based on principles of content analysis [12]. Codes were generated from the interview data and then systematically applied to identify themes and patterns. For English-speaking participants, HT read the transcripts, conceptualised, and coded all components of the qualitative data relating to dementia-related care services into concepts identified inductively; new codes were created when required. A research assistant supported the coding of transcripts in French. Similar services were grouped into themes and subthemes. Clinical experts with experience caring for the patient population from each population centre were consulted in the event of uncertainty relating to coding or development of themes or subthemes.

Three clinical experts (including JS and WD) from each location reviewed the services included in each theme and subtheme to ensure that services were classified appropriately. Time frames from the vignette fitting the necessary provision of the services were derived from the Canadian consensus statement and determined in consultation with the clinical experts. Themes and subthemes were subsequently quantified using quantitative content analysis to examine the association between study population centres (i.e. Calgary, Edmonton, and Ottawa) and services during guideline-concordant stages of the disease [10]. Chi-square tests (or Fisher’s exact test as appropriate) were used as the main statistical analysis. The association between type of healthcare provider and each theme was also examined. All analyses were undertaken using Stata 11 [13].

## Results

### Participant characteristics

Thirty-four healthcare providers from Calgary (*n* = 12), Edmonton (*n* = 12), and Ottawa (*n* = 10) participated, including family physicians (*n* = 12), specialists (*n* = 13), and case managers (*n* = 9). Thirty-one were English-speaking and three were French-speaking.

### Overarching themes of dementia-related services

**Fig. 1:**
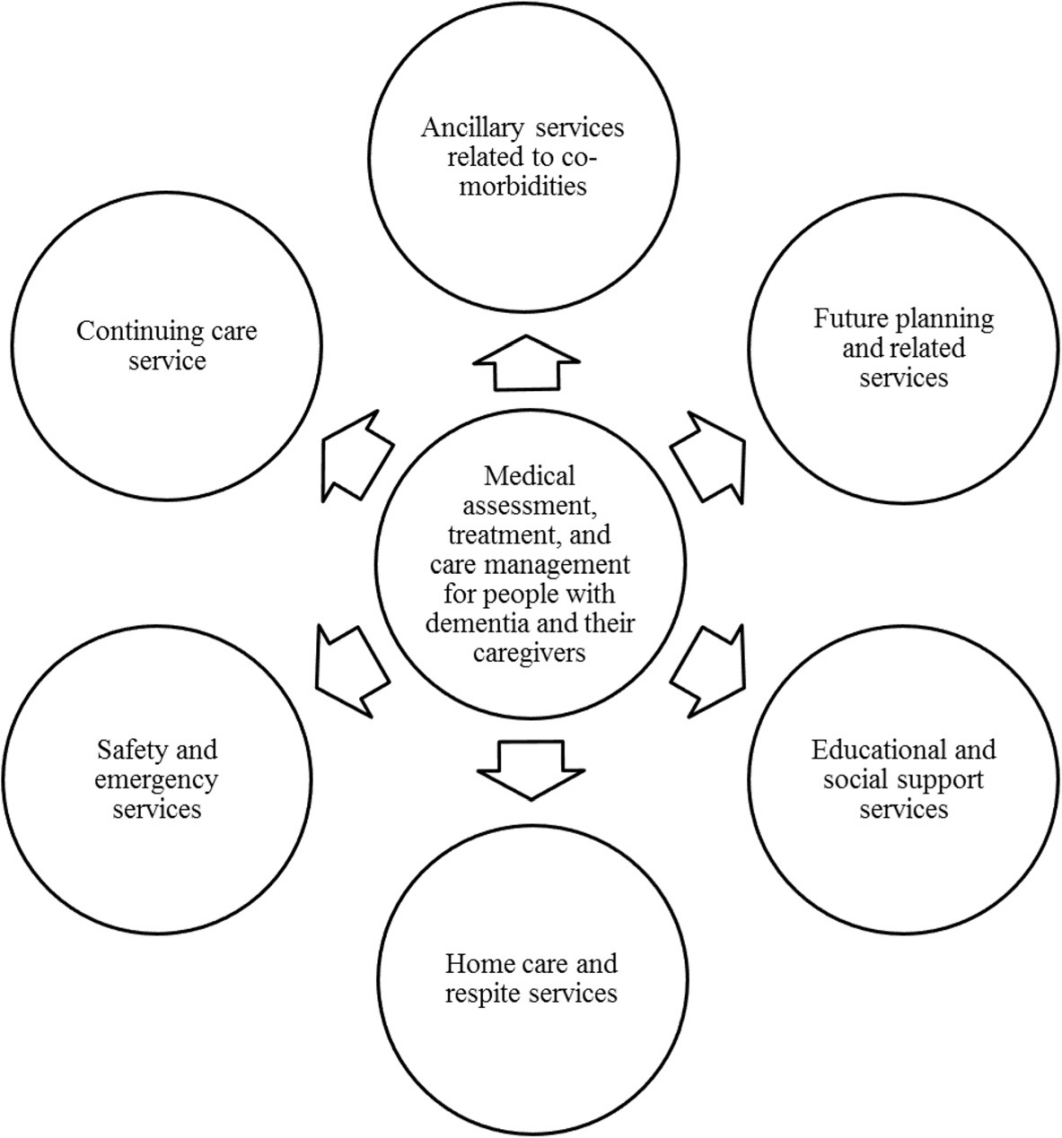
Overview of dementia-related care services. *Note*. Medical assessment, treatment, and care management includes risk factors, diagnosis, and behavioural and psychological symptoms of dementia; it brings together all the dementia-related types of services located on the periphery. Safety and emergency services relate to transportation, falls, wandering, and contingency services. Continuing care services provide increased care at a hospital or housing alternative. See main text for details on future planning, educational and social support, and home care and respite services

Review of our data identified seven overarching themes of dementia-related services (Fig.1). Frequently identified services provided in the community setting were compared across population centres; a limited number of participants practiced in long-term care or in an in-patient setting, hence only services specific to the community setting were further examined. These related to future planning and related services, educational and social support services, and home care and respite services. Although providers in our sample consistently identified educational and social support services within the recommended time frame according to national recommendations, provision of the other service types were more variable. See Table 2 for a summary of services compared across study sites and providers.

**Table 2:** Provision of guideline-concordant dementia care

Type of care	Future planning support	Educational and social support	Home care and respite services		
			Informal IADL/ADL support	In-home support services	Day program services
Recommended Time Frame					
Earliest time point	T1: warning signs of MCI	T1: warning signs of MCI	T1: warning signs of MCI	T5: dementia diagnosis	T5: dementia diagnosis
Latest time point	T8: functional impairment	T9: caregiver stress	T9: caregiver stress	T9: caregiver stress	T9: caregiver stress
Setting	*n* (%)	*n* (%)	*n* (%)	*n* (%)	*n* (%)
Calgary	11 (91.7)	12 (100.0)	6 (50.0)	11 (91.7)	12 (100.0)
Edmonton	7 (58.3)	12 (100.0)	9 (75.0)	9 (75.0)	11 (91.7)
Ottawa	3 (30.0)	10 (100.0)	7 (70.0)	7 (70.0)	6 (60.0)
*p*-value	0.012	−	0.403	0.409	0.023
Provider					
Family physician	5 (41.7)	9 (100.0)	8 (66.7)	10 (83.3)	9 (75.0)
Specialist	9 (69.2)	12 (100.0)	6 (46.2)	8 (61.5)	11 (84.6)
Case manager	7 (77.8)	13 (100.0)	8 (88.9)	9 (100.0)	9 (100.0)
*p*-value	0.189	−	0.117	0.083	0.277

*ADL* activities of daily living, *IADL* instrumental activities of daily living, *LTC* long-term care, *MCI* mild cognitive impairment, *T* time point based on the vignette (Table 1)

### Future planning and related services

While patients retained decision-making capacity, eleven participants in Calgary (91.7 %) reported accessing future planning services, including medical and financial decision support services eliciting patient wishes and preferences. This proportion was significantly greater than in the other sites (Edmonton, 58.3 %; and Ottawa 30.0 %), *p* = 0.012. Family physicians had the lowest proportion of participants that identified future planning services (41.7 %), though the differences were not statistically significant (*p* = 0.189). Although not recommended, four participants (11.8 %) identified this type of service for the first time when the patient was in a late stage of dementia. Among participants that mentioned this service, personal directives and power of attorney were similarly identified. Both Edmonton and Calgary participants identified Advance Care Planning, while providers in Calgary specified the Goals of Care Designation Order.

### Educational and social support services

All participants, regardless of population centres and type of provider, identified the Alzheimer Society and other formal services with educational workshops and support groups, including caregiver-training programs, within the recommended times. However, participants from Calgary identified five unique educational and social support programs not mentioned anywhere else. For example, the Family Caregiver Centre provides access to social workers for education, support, and liaison to healthcare and community services; the Kerby Centre and Living Well programs administer a range of activities, education classes, and prevention services; and the Memory Plus Program serves people with mild dementia and their caregivers to prevent caregiver fatigue. Participants from Calgary also mentioned these services earlier in the disease trajectory compared to the other sites, e.g. 66.7 % of participants from Calgary identified this service by the time there was early recognition of mild cognitive impairment (time point 2), while only 16.7 % participants in Edmonton and 10.0 % participants in Ottawa mentioned this service (*p* = 0.006).

### Home care and respite services

#### Informal support for basic and instrumental activities of daily living

A higher proportion of participants in Edmonton (75.0 %) made timely mention of support by family or friends for basic and instrumental activities of daily living, including emotional and practical support, compared to those in Calgary (50.0 %) and Ottawa (70.0 %) though the proportions were not statistically different (*p* = 0.403). In particular, case managers (88.9 %) appear to be important in mobilizing this resource, while fewer physicians considered it (66.7 %), though the proportions were also not statistically different (*p* = 0.117).

#### In-home support services

In-home support services consist of home care (e.g. personal care) and respite services provided in the patient’s home. Guidelines indicated that these services should be mentioned to support caregiver and patient by a moderate stage of dementia. Our respondents called upon various types of organizations to provide these services, including non-profit, governmental, and private entities. Among participants mentioning in-home support services, a key resource was Meals on Wheels. Calgary and Edmonton participants similarly mentioned Home Care for a variety of tasks (including in-home respite, personal care, housekeeping, and medication monitoring), while Ottawa participants identified resources unique to their location. Though non-significant, greater proportions of participants from Calgary (91.7 %) identified in-home support services by the guideline-recommended time point in the vignette compared to other sites (75.0 % in Edmonton and 70.0 % in Ottawa, *p* = 0.409). Similarly, though non-significant, greater proportions of case managers (100.0 %) and family physicians (83.3 %) identified in-home support services by the recommended time (*p* = 0.083).

#### Day program services

Day program services are integral to dementia care in the community as they provide socialization for people with dementia and respite for their caregivers. Almost all participants from Edmonton (91.9 %) and all from Calgary (100.0 %) identified day program services within the recommended time frame compared to only 60.0 % of participants in Ottawa (*p* = 0.023). Overall, the majority of the three types of providers identified day program services (i.e. 100.0 % of case managers, 84.3 % of specialists, and 75.0 % of family physicians, *p* = 0.277).

## Discussion

We found variation between three major Canadian cities in patterns of dementia-related care across the disease trajectory, specifically for the provision of future planning and day program services. However, differences were not substantial across types of providers (family physicians, geriatric medical specialists, and dementia case managers) for community services supporting the patient and caregiver prior to admission into long-term care. A key commonality was observed among participants when providing educational and social support services (e.g. the Alzheimer Society) in a timely manner in concordance with the Canadian consensus statement on dementia care.

Providers should discuss future planning by the time patients are in a moderate stage of dementia (i.e. time points 5 to 8), rather than late stages of the disease, as the patient may still retain decision-making capacity despite their increased need for support in their activities of daily living and experience with behavioural and psychological symptoms. We found that participants from Calgary generally reported accessing future planning services earlier, by the latest time in the vignette according to guidelines, and proportionately more than other sites. This may be attributable to the presence of resources in Calgary dedicated to future planning (e.g. Calgary-specific Advanced Care Planning and Goals of Care services) that have heightened the awareness for initiating these discussions. However, given that Alberta Health Services manages care for the entire province, future investigation is required to understand this provision of services in Edmonton. In Ontario, research has previously reported that family physicians seldom use advanced directives and that they are more likely to arrange them for patients that are terminally ill [14]. Despite current evidence to suggest that future planning improves end of life care, reduces stress, anxiety [15], and patient preferences for early initiations of these discussions [16], providers may delay the discussion due to their discomfort [17], perceived prerequisite of a stronger patient-physician relationship, and fear of jeopardizing their patient’s hope [18]. Our findings suggest that providers should be further educated on improving the timing and frequency of future planning, particularly in Edmonton and Ottawa.

Community services should be provided by a moderate stage of dementia to share the caregiving role. For home care and respite services, we found that case managers, in particular, appeared to be important in mobilizing this group of resources. This is important, as case managers have been demonstrated to delay long-term care [19]. We also found lower proportions in timely access to day program services among participants in Ottawa compared to Calgary and Edmonton, which may reflect differences in practice (e.g. timing and process of service implementation), availability, and/or perception of necessity of services between Ontario and Alberta. The Alzheimer Society and similar services were observed to be important resources, as providers from all sites tended to identify them by the recommended time. This type of service appeared to be regarded as integral to dementia care and may facilitate timely access to local resources [20]. Improvements may be indicated to ensure that patients are aware of their local resources, as delayed referral to home care has been reported to impact caregiver health [8]. Further, increasing awareness and acceptance of community services is key to patient satisfaction [9] and could address caregiver service utilization issues [7].

For a person with dementia living in Canada with universal healthcare coverage, this study illustrates a diversity of services in place for their care, particularly in the community setting. However, despite being in large population centres with a high intensity of care, this study suggests that access to particular services at the time when they require it may depend on the specific centre that they reside in. Although this may be attributable to the nature of the Canadian healthcare system, where each province (e.g. Alberta and Ontario) is responsible for the organization and delivery of healthcare services [21], variation was also found within province (i.e. Edmonton versus Calgary within Alberta). While these results require further investigation, these variations may have implications on clinical outcomes (e.g. patient quality of life and caregiver burden), and reflect the level of preparedness of our healthcare system to care for an aging population living with progressive chronic diseases. To assist in the timely provision of necessary services, an informational resource on guideline-concordant services (e.g. as part of a clinical pathway) could be developed and tested for providers caring for patients with dementia in the community. This information could be standardized across various centres and adapted to local resources, such as those highlighted in this study, to support providers as part of their continuing professional development.

This study used a longitudinal vignette as the basis for standardizing comparison of service access behaviours for people with dementia in three Canadian centres, among three sets of providers, to assess the landscape regarding services provided for patients with dementia. We acknowledge the relatively small numbers of participants in each of the groups, which may explain some of the non-significant results presented in this study. Also, we recognize the limited representation of case managers in Ottawa, which may cause selection bias and decrease the proportion of identification from case managers that are within the scope of their practice (e.g. future planning, informal social support, home care, and respite services). Because the vignette was designed to capture the typical characteristics of dementia cases most commonly seen in primary care settings, our findings focus on this type of patient. Also, the use of a vignette for exploring clinical management decision-making may be criticized as being different from a study examining actual events. Nevertheless, it is a mechanism for uniformly presenting the same case to all study participants, which functions to reveal their awareness of services, the points in the disease trajectory when they would consider accessing them, and the relationship of those time points to guideline recommendations. Hence, we believe the method has considerable merit and provides evidence for the areas requiring improvement and future investigation. The tools and knowledge gained from this study support the need for a larger inquiry to examine timely service provision, engage a more representative sample of case managers, and refine the understanding of major services. Also, future research is warranted in examining barriers and facilitators to provision of future planning in Edmonton and Ottawa, and day program services in Ottawa.

## Conclusions

This study indicates that there are variations between major Canadian cities in patterns of health services delivery across the disease trajectory for patients with dementia. We provide an assessment of the range of services indicated by national guidelines for dementia care to be calibrated with verifiable provision of services in future work. These results can inform a future national dementia care plan to reduce the variability of services provided for patients and their caregivers with dementia. Also, our findings have implications for continuing medical education and professional development, particularly for services provided inconsistently among Canadian healthcare settings.

### Consent to participate

Written or verbal consent for participation in the study was obtained from participants prior to the interview. This study was approved by the Conjoint Health Research Ethics Board of the University of Calgary (E-22186) and the Council of Research Ethics Board of the University of Ottawa (H04-09-01).

### Consent for publication

Not applicable.

### Availability of data and material

The interview transcripts are confidential and therefore not publically available.

## Additional file

Additional file 1: Interview Guide. At each time point of the vignette (Table 1), the interviewer asked the following questions. Interviewers were permitted to ask follow-up questions for clarification. 1) What action would you take if the patient was presented to you at this stage with this history? 2) Are there any other resources you would consider at this stage? (DOCX 11 kb)

## Abbreviation

CI: confidence interval
