# Supplementary material for: Provision of dementia-related services in Canada: a comparative study
Source: BMC Health Serv Res. 2016 May 17;16:184. doi: 10.1186/s12913-016-1435-1 (PMC4869270; doi:10.1186/s12913-016-1435-1)
Supplement: Additional file 1: — Interview Guide. At each time point of the vignette (Table 1), the interviewer asked the following questions. Interviewers were permitted to ask follow-up questions for clarification. 1) What action would you take if the patient was presented to you at this stage with this history? 2) Are there any other resources you would consider at this stage? (DOCX 11 kb) [file 12913_2016_1435_MOESM1_ESM.docx]

**Additional File 1. Interview Guide**

At each time point of the vignette (table 1), the interviewer asked the following questions. Interviewers were permitted to ask follow-up questions for clarification.

1) What action would you take if she presented to you at this stage with this history?

2) Are there any other resources you would consider at this stage?
